# Sustaining e-health innovations in a complex hospital environment: learning through evidence

**DOI:** 10.3389/fdgth.2024.1346085

**Published:** 2024-04-30

**Authors:** Mirou Jaana, Erika MacPhee, Heather Sherrard, Mark Walker

**Affiliations:** ^1^ Telfer School of Management, University of Ottawa, Ottawa, ON, Canada; ^2^ University of Ottawa Heart Institute, Ottawa, ON, Canada; ^3^ Faculty of Medicine, University of Ottawa, Ottawa, ON, Canada; ^4^ Ottawa Hospital Research Institute, Ottawa, ON, Canada

**Keywords:** e-health, virtual care, digital health, innovation, hospital, telehealth, telehome monitoring, interactive voice response

## Abstract

Implementing and sustaining technological innovations in healthcare is a complex process. Commonly, innovations are abandoned due to unsuccessful attempts to sustain and scale-up post implementation. Limited information is available on what characterizes successful e-health innovations and the enabling factors that can lead to their sustainability in complex hospital environments. We present a successful implementation, sustainability and scale-up of a virtual care program consisting of three e-health applications (telemedicine, telehome monitoring, and interactive voice response) in a major cardiac care hospital in Canada. We describe their evolution and adaptation over time, present the innovative approach for their “business case” and funding that supported their implementation, and identify key factors that enabled their sustainability and success, which may inform future research and serve as a benchmark for other health care organizations. Despite resource constraints, e-health innovations can be deployed and successfully sustained in complex healthcare settings contingent key considerations: simplifying technology to make it intuitive for patients; providing significant value proposition that is research supported to influence policy changes; involving early supporters of adoption from administrative and clinical staff; engaging patients throughout the innovation cycle; and partnering with industry/technology providers.

## 1 Introduction

Healthcare organizations are often slow to innovate, and it is estimated that 30%–90% of all innovation implementations fail (1). Innovating within the healthcare system involves the implementation of various new ideas/concepts (e.g., those related to service delivery), which is often a desirable change for improving the quality of care albeit the disruption and risks that it may bring (2). Information technologies (IT) represent innovations that may be leveraged by healthcare organizations to improve patients' care, reduce costs, and enhance the delivery of health care services (3). Nevertheless, despite their potential, IT innovations have been historically challenged in the health care environment and the rate of failure of IT projects is high (4). Compared to other sectors, the healthcare industry has lower levels of IT innovation, which is often attributed to the particularities and challenges faced within this environment (e.g., concerns related to ethics, privacy, security, and accountability) (5). Healthcare organizations are sometimes resistant to change, due to uncertainty and distrust toward innovations, organizational culture, and structural factors (e.g., organizational characteristics, resources, etc.) (6). Furthermore, the implementation of IT solutions often necessitating the changes in clinical and administrative workflows, thus buy-in from stakeholders to ensure successful technology implementation (7).

The spread, sustainability, and scale-up of healthcare innovations are often limited, with the majority involving pilot projects or single organizations (8). “Spread” is a term that is used to describe the implementation of an innovation and its adaptation to a new setting, whereas “sustainability” and “scale-up” refer to the state when it becomes part of routine practices and when it reaches all relevant recipients who can benefit from it (8). IT innovations are often abandoned before reaching sustainability and scale-up as healthcare organizations often lack financial resources, face technical challenges (e.g., lack of interoperability), and have limited capacity to support these IT solutions (4, 9). Thus, like other innovations, their implementation often fails to consider their long-term use, and the new practice accompanying these innovations falls short of being integrated into the routine activities and workflows of clinicians (10). Existing literature has emphasized the need for continued research on spreading and sustaining innovations, particularly work that shares the lessons learned for system level changes (11), and called for studies that can help better understand how health innovations can be sustained in practice (10).

Informed by document reviews and the input of representative clinical stakeholders (i.e., two physicians and four nurses), we present the case of a Cardiac Virtual Care (CVC) program, including three successful e-health applications, at a major specialty hospital in Canada, which can serve as a benchmark for other hospitals and inform future e-health innovations implementation. First, we present an overview of existing literature on spreading and sustaining innovations in healthcare and describe the history and evolution of the e-health applications used in this CVC program. In the subsequent sections, we provide a narrative of the multiple factors that influenced this complex initiative, identify how the complexities and challenges were mitigated, and discuss the role of practice-research partnerships in supporting this innovation.

## 2 Literature review

The sustainability of innovations is particularly important within the healthcare sector to ensure that the invested resources lead to benefits for the respective organizations and improve patients' care (12). In their scoping review, Côté-Boileau and colleagues discussed the reasons that may contribute to the challenges to innovate in healthcare organizations including high levels of inertia, limited availability of resources, and unpredictability of the environment in which the organizations operate (8). They presented evidence on facilitators that have been discussed in the literature as enabling healthcare innovations (e.g., leadership and management support, timing, collaboration among and within jurisdictions, context) and recommended that future research report on challenges and lessons learned when spreading, scaling, and sustaining innovations within healthcare organization to inform future initiatives in similar settings (8).

More recently, Gusmão Louredo et al. conducted a systematic review to examine the complexities within the hospital environment that can facilitate or hinder innovation (12). They discussed the heterogeneity of hospital services and the diversity of departments and workflows that an innovation must adapt to, which make innovations challenging, and emphasized the importance of the perceived relevance of technology and individual healthcare professional's resistance to change (12). At the organizational level, continuous technical and financial support and ongoing training were considered essential to sustaining innovations over time (12).

Various frameworks and models have been proposed to conceptualize the sustaining and scaling-up of innovations in healthcare (4, 13–15). Among these, a comprehensive model proposed by Greenhalgh and colleagues i.e., the *non-adoption abandonment, scale-up, spread, and sustainability (NASSS) framework* for non-adoption and abandonment of technologies, discussed the challenges to scaling-up, spreading and sustaining technologies in health and care organizations (4). In this model, the authors emphasized the relevance of patients' conditions, technology, value proposition, key stakeholders, and organizational-level factors in supporting the embedding and adaptation of technologies over time. They also discussed the inherent common challenge associated with the abandonment of technologies, and the limited information on real cases that demonstrate the ingredients to the successful scale-up of IT innovations (4).

The NASSS framework has been applied within hospital settings to better understand the barriers and facilitators toward technology implementation (16). For example, in a scoping review by Abell and colleagues mapped the barriers and facilitators towards implementing clinical decision support systems (CDSS) in hospital settings using the NASSS framework (16). They found 44 studies which revealed that the implementation of CDSS often had little perceived relative advantage for clinicians in the hospital setting, and many of the reported barriers were mostly aligned with the condition/context (e.g., clinical context, inability to adapt the CDSS systems, etc.), the technology (e.g., limitations in the technical features of the solution, information redundancy, etc.), and the adopter (e.g., professional autonomy of clinicians, perceived complexity, usefulness, and usability of the technology, etc.) domains of the NASSS framework (16). In this paper, we present the case of a CVC program IT innovation, which includes three e-health applications, and its evolution at a Canadian hospital, discuss its sustainability and critical success factors grounded in the NASSS framework, and provide lessons learned that may serve as benchmarks for other IT innovations and settings.

## 3 Context

The University of Ottawa Heart Institute (UOHI) is Canada's largest heart health centre, delivering care to over 210,000 patients annually (17). It specializes in the treatment and prevention of heart disease for patients from rural and urban settings in the Ottawa region as well as other areas across the country. Over 25 years, the UOHI developed the CVC program, which represents an IT innovation that consists of virtual care services implemented through three e-health applications aiming to support the overall management of heart disease. The program was initiated to provide nursing support and address the increasing need of cardiac patients who require assistance with medication management, fluid volume regulation, vital sign monitoring and patient education (18). Its initiation as a pilot innovation project began with minimal funding in the early 2000's, but was successfully sustained over time, and grew to permanently integrate the three e-health applications in the process of care and services delivery model to patients at the UOHI.

## 4 History and evolution

**Figure 1:**
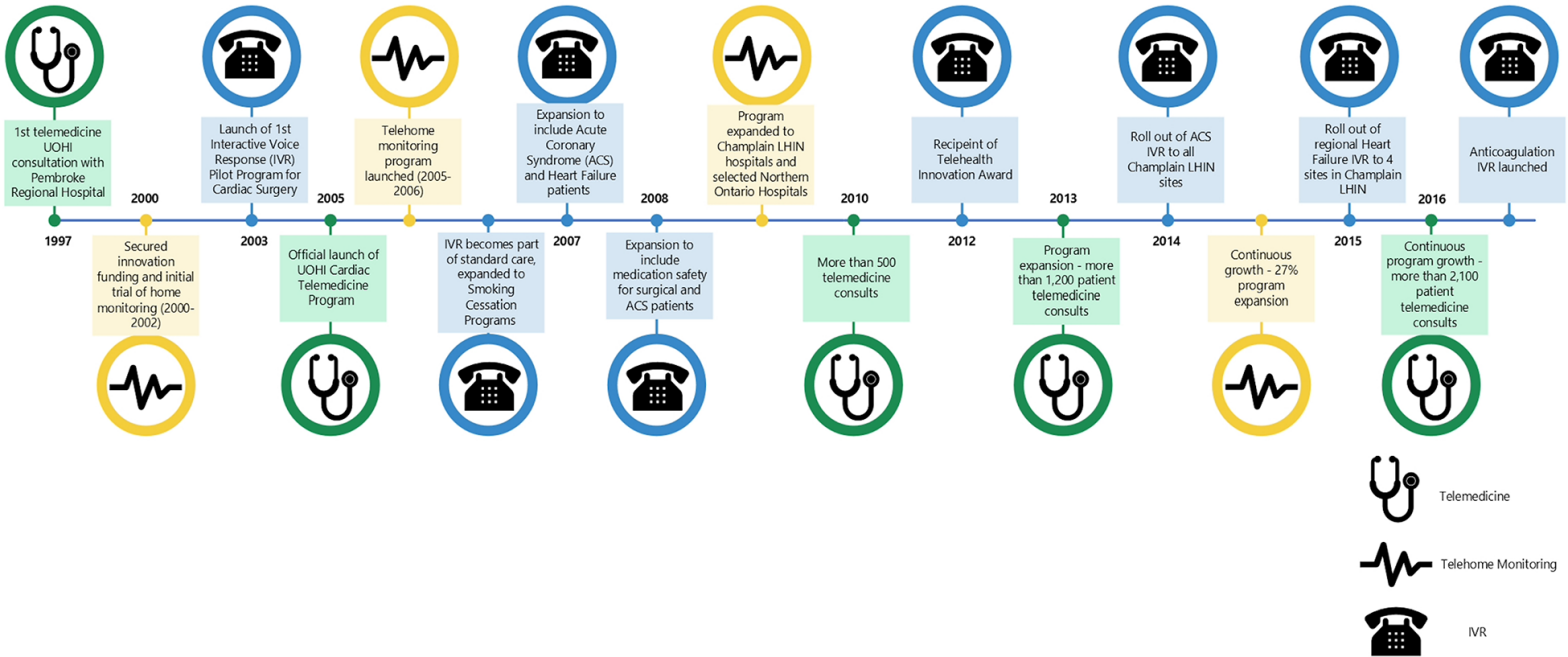
The e-health journey at the University of Ottawa Heart Institute.

The e-health journey at the UOHI started in 1997 and continues until today (Figure 1), focusing on surgical patients who are now able to be discharged earlier than in the past given the technology-supported follow-up, and then branching out into services for heart failure (HF) and acute coronary conditions. As shown in Figure 1, the innovation process passed through stages that demonstrated the relevance and value proposition of each technology to the different cardiac patient conditions and was shaped by stakeholders' engagement and key enabling organizational factors. Subsequently, three e-health applications became integrated in the care delivery for patients receiving care at the UOHI: Telemedicine (TM), Telehome Monitoring (THM), and Interactive Voice Response (IVR).

The CVC program originated as an innovation to meet the healthcare needs of the hospital's population and evolved into an established e-health unit, providing three types of e-health services that cater for the varying needs of cardiac patients in the capital of Canada and the region (Figure 1). It was established in 2000 with initial funding from the Richard Ivy Foundation and a grant from the Change Foundation that supported a large-scale randomized control trial with 249 HF and angina patients. The trial compared the impacts of a 3-months THM care post discharge with usual care and found that the former significantly reduced the number of hospital readmissions and hospital days (19). Patients also expressed high level of satisfaction with this technology, better quality of life, and improved functional status (19).

Based on these early positive results, permanent funding was provided by CareConnect (former telemedicine network servicing eastern Ontario that merged with two other networks to form the Ontario Telemedicine Network in 2006). Additional 40 home monitors were purchased and the formal THM application was launched in 2005. In 2006, the IVR technology became part of the standard care for cardiac surgery patients and expanded to HF and Acute Coronary Syndrome (ACS) patients. In 2008, additional funding from the Champlain Local Health Integration Network (LHIN) enabled the expansion of THM to most hospitals in the region (20). Patients at these hospitals received training on how to use the THM equipment from local nurses; the data were transmitted to the CVC program at the UOHI that coordinated data sharing with community physicians (20).

The number of patients enrolled in the CVC program, through the three digital modalities of e-health applications, grew over time. The cardiac telehealth metrics pre-pandemic (2018–2019) showed that 12,030 patients were enrolled in the CVC program at the UOHI: 7978 patients in TM, 352 patients in THM, and 3700 in IVR [Cardiac surgery—1381, Acute Coronary Syndrome (ACS)—2183, and HF—136]. In 2022, three years into the pandemic, there were 325 THM patients, 4244 IVR patients using these technologies. The TM application was amalgamated with a larger system. In June 2019, with the UOHI joining five other health care organizations in the Atlas alliance implementing a new hospital information system (HIS) from Epic (21), TM consultations have since decreased to 412 in 2022 as all rehabilitation offerings are now completed via an application in the new e-chart-Epic Zoom.

## 5 Three technologies in One innovation

The CVC program represents an innovation consisting of a participant-facing digital platform for cloud-based population health management (22). The features include assessments and preferences, personal care plans, trackers, progress reports, integration with fitness devices, reminders, circle of care invitations, online peer support groups, and group challenges. Patients who are enrolled in the CVC program have diverse cardiac health conditions including HF; post -operative cardiac surgery; post heart attack; arrythmias (irregular heart rhythms). Three e-health applications (i.e., TM, THM, IVR) are currently used, and patients are matched at discharge to a technology according to their needs.

### 5.1 Telemedicine

The TM application, originated as part of the Healthcare and Education Access for Remote Residents by Telecommunications (HEARTT) project spearheaded by UOHI researchers in the mid-late 1990s (23). At its inception, the technology connected cardiologists at the UOHI with residents living in three rural towns that were located within a distance of 45-minute drive to almost 1000 kilometers away from UOHI (23). Several partners worked together with the UOHI to facilitate this undertaking including:

- The Government of Ontario provided $2 million in funding.
- The federal government through Industry Canada provided assistance for establishing satellite networks such as those required in tests sites that lacked broadband infrastructure at the time (23).
- Four private technology corporations provided the technological equipment that facilitated data transfer from the sites to the consultants (23).

The HEARTT pilot project demonstrated early success of the TM strategy through the positive reviews obtained from surveyed patients and cost savings associated with reductions in emergency transfers and shortened hospital stays (23). However, physicians in the HEARTT project were unpaid and provided consultations pro-bono (23). The positive findings on one hand, and the reimbursement challenges on the other hand, helped spur the creation of the Eastern Ontario Telehealth Network in 2001 that received funding from the provincial government (24). This service is now delivered to patients via the Ontario Telemedicine Network (OTN) (25). Eligible patients travel to a designated nearby hospital or community health centre, instead of having to travel to the UOHI for their appointment. These sites are equipped with video conferencing equipment and medical equipment, and are staffed by a nurse allowing consultants at the UOHI to provide a full suite of care to them (25). TM delivered via the OTN has demonstrated positive impacts related to reducing travel costs, improving patient satisfaction, decreasing hospital admissions, supporting provider efficiency, and increasing access to services (26). During 2011–2012, delivering telemedicine through the OTN was estimated to have saved patients 130 million miles of travel (approximately 23 million litres of gasoline), and about $45 million in travel cost subsidies for the Ontario government (26). During the same period, 2378 TM consultations for stroke were estimated to have saved $3.5 million in healthcare systems costs due to timely administration of tissue plasminogen activator for 722 individuals, which decreased the number of hospitalizations and amount of required nursing home care (26).

### 5.2 Telehome monitoring

THM represents an e-health application that is based on an acute intervention model used to manage the health condition of diverse patient populations. It enables early detection of health deterioration and prompts timely intervention by health professionals (27).

Patients at the UOHI who require daily monitoring are shown how to use the THM equipment, which is provided to them at no cost, before going home. The technology uses recorded voice prompts and provides simple, clear instructions to capture vital signs and other health information in a non-invasive manner, which are then transmitted automatically to a central station at the hospital. The data are reviewed regularly by expert cardiac nurses, and patients are called for further assessment and intervention as needed.

THM offers an effective patient management approach that can be used to support patients with a broad range of chronic diseases (28–34). Research has shown that this technology can have positive impacts on patients living with HF (30–34). Evaluation of THM use at the UOHI reported a significant reduction in hospital admissions and length of stay, and an improvement in quality of life for patients with angina (19). A more recent study comparing THM use by HF patients in rural vs. urban areas revealed similar utilization pattern and no significant differences in process and outcomes measures among the two groups, further confirming its benefits to various groups of patients (35).

### 5.3 Interactive voice response

IVR uses a regular phone line to transmit clinical information from home to a central station located at the UOHI. The following IVR applications offered under Clinical Services support the follow-up of patients with diverse conditions after hospital discharge:

1. Cardiac surgery—For symptoms screening of patients discharged following open-heart surgery until seen by a surgeon. Call frequency = Days 3,10 + Weeks 1,2
2. ACS—To maintain patients on best practice guidelines including questions regarding symptoms, medication adherence, adoption of health behaviors such as smoking cessation and participation in cardiac rehabilitation. Call frequency = Days 2,7 + Weeks 1,3,6,9,12
3. HF—To promote self-care education, symptom screening, adherence to HF medications, and offer mail-out information. Call frequency = Days 2,7 + every 2 weeks for 3 months
4. Diabetes—To identify undiagnosed diabetes (HbA1C ≥ 6.5) and follow-up patients with known diabetes post hospital discharge after admission for cardiac surgery, ACS, or HF.

The automated calling has an algorithm of clinical questions, which mimics a health professional's assessment. Patients are called at regular intervals and asked to respond to various questions assessing their condition and behavior (answering “yes” or “no” or by using the phone keypads). A nurse is flagged to call a patient as needed, depending on the responses provided. Areas of assessment include occurrence of symptoms (ankle swelling, difficulty breathing), medication compliance (e.g., Betablocker, ACEI/ARB), weight gain, lifestyle choices (e.g., eating/drinking, walking). In addition, educational material is shared with the patients who indicate interest in getting more information about how to better manage their condition.

Prior assessment of IVR use at the UOHI reported better compliance and less adverse events for patients with Coronary Artery Bypass Graft (36), and positive outcomes associated with this technology use among patients with ACS (37). A more recent study on IVR use among HF patients also reported an increase in medication adherence and a decrease in symptoms occurrence, weight gain and readmission rates over a 12-week period (38).

## 6 Discussion

The CVC program uses an acute Intervention Model and is designed to offer services to patients similar to those they would receive in a hospital setting. Self-care education is also provided and includes information on the benefits of compliance with daily weight, salt & fluid restriction, medication education and symptom management.

### 6.1 Sustainability and critical success factors

What differentiates the CVC program at the UOHI from other e-health programs is that it is a “nurse-run” program with available medical leads when needed. Expert cardiac registered nurses are available to provide care between medical visits and deal with issues as they arise. This is particularly important considering resource constraints, limited capacity, and tight schedules for in-person appointments with medical professionals. There are no fees for patients who join the CVC program. TM is funded by the Ontario Ministry of Health and Long Term Care (MOHLTC) and physicians bill the Ontario Health Insurance Plan (OHIP) directly (39); THM and IVR, on the other hand, are funded by the UOHI through its operating budget, which was realized through the cost savings associated with the reduction in readmissions at the UOHI (40). For example, the average cost of a daily stay for a patient with HF at UOHI is approximately $1000, with an average length of stay of approximately one week. As the CVC program reduced admissions by 54%, this covered the operational budget for the remote monitoring team (i.e., 5 full-time staff members) and approximately $250,000 for monitoring fees and equipment maintenance. The challenges usually encountered in relation to the abandonment and non-adoption of technologies were not evident in the CVC program context. According to Greenhalgh (4) and Greenhalgh and Abimbola (15), the higher the complexity of the program being implemented along the seven domains identified in the NASSS framework and described earlier, the less likely it is that the technology would achieve sustained adoption. Nevertheless, in the case of the CVC program, despite the relative complexity of the environment in which it was implemented, there was a sustained adoption of the three e-health applications as evidenced by their integration into the usual care process.

**Figure 2:**
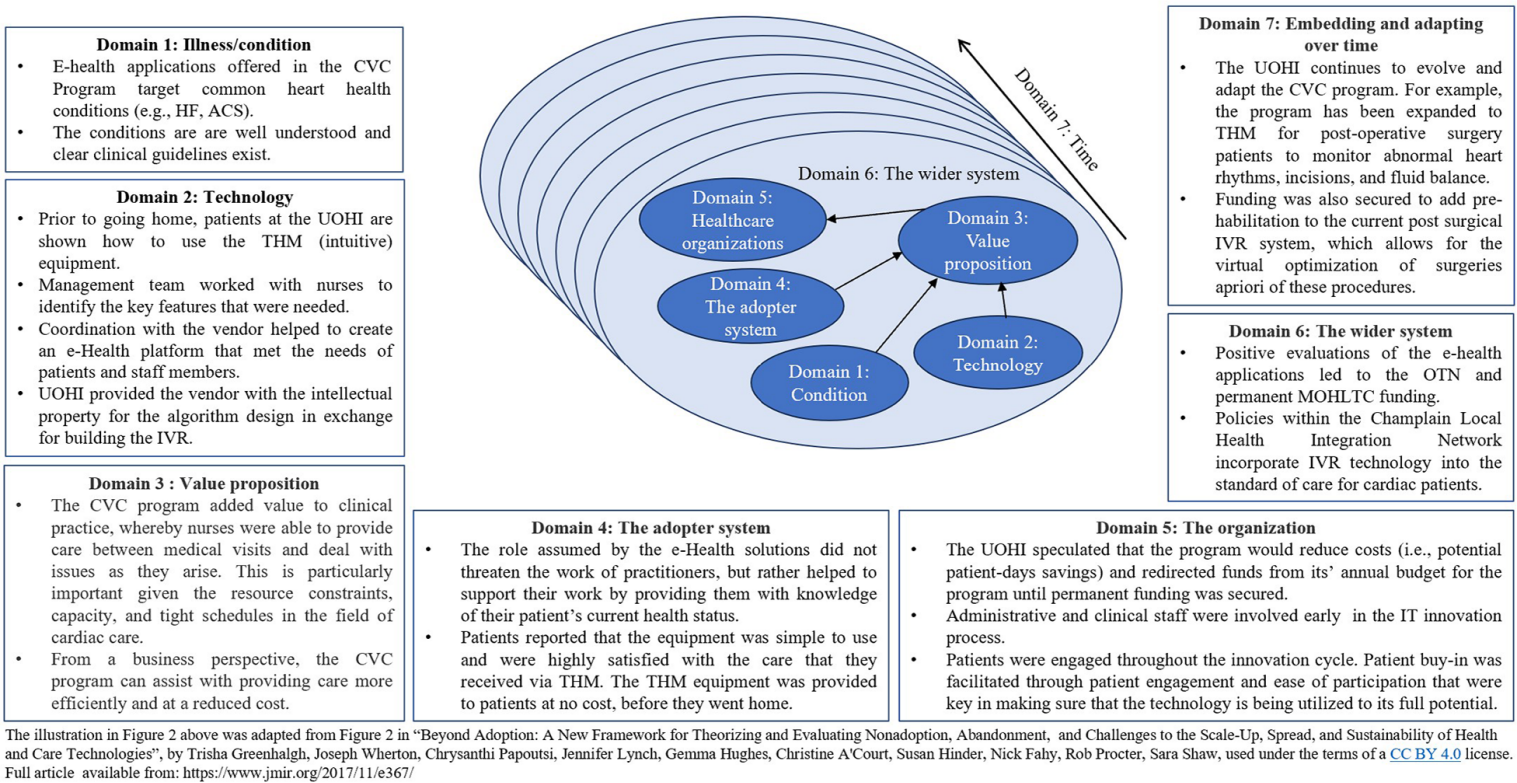
Lessons learned from the CVC mapped to the nonadoption, abandonment, scale-up, spread, and sustainability (NASSS) framework (4).

The sustainability and success of the CVC Program is multifactorial (Figure 2), as per the input of the six stakeholders and the documents review. The e-health applications offered target common heart health conditions (e.g., HF, ACS) that are well understood with clear clinical guidelines. Feedback obtained from nurses on the factors that influenced the success of the CVC program emphasized that, while standardized guidelines for treating cardiac patients exist, the nurses handle each patient on a case-by-case basis. They adopt a systems approach to providing care (e.g., when changing one medication, considering how it may affect other chronic illnesses the patient has). Although they acknowledged the effectiveness of the technologies used, they also highlighted that it is important to have experienced nurses on board who are comfortable making difficult decisions when presented with the data being transmitted to the UOHI from patients through these technologies.

Staff training is another factor that was critical to the sustainability and success of the CVC program. The training offered to all staff consisted of multiple approaches including one-to-one training, rounds on the unit (e.g., providing information and contacts to those working if they needed help), and mentoring between staff members. While the nurses indicated that there was a big learning curve for the technologies, role playing activities where the nurses brought the equipment home to test as a mock patient were also utilized. This helped to prepare them for questions that were asked by the patients.

The physicians indicated that the underlying technologies used in the early stages were intuitive and simple to use (e.g., THM devices attached to a weighing scale/blood pressure measuring cuffs/pulsometer, using telephone outlets for data transmission, with intuitive on/off buttons and a small screen avoiding confusion). Non-compliance was seldom an issue, and more often, patients wanted to continue to be monitored following their discharge from the CVC program, as they found comfort in knowing that someone was there to watch over them and help when needed. Patient engagement and ease of participation are key in making sure that the technology is being utilized to its full potential. With respect to THM, patients are trained in the technology use before they are discharged from the hospital, and the intuitive nature of the technology/device used enabled successful adoption.

The initiation of THM was supported by research and innovation funding, which were used to demonstrate early on the safety and feasibility of this new model of care. Once established, the UOHI carved funding from its annual budget based on potential patient-days savings and directed these financial resources toward growing the program. Based on the evidence of success, permanent MOHLTC funding was secured.

The input from the clinical staff members (*n* = 6) indicated high buy-in for the CVC program because of their ability to remain connected with their patients, which they believe is vital to providing care, especially given the acute and immediate needs of the patients they see. Staff and patients (“adopters”) drove the sustainability and success of the CVC program through their “ownership” of these technological innovations. They were involved in the choice and development of the respective technologies. The management team worked alongside the nurses to identify the features to include in the systems, and the team coordinated with the vendor to create an e-health platform that would support patient and staff needs. Easy access to expert clinicians was essential in the process. A clear process to identify and pair patients with the right e-health application also led to more successful outcomes. Having a health care provider who is knowledgeable of the patient's health status is essential, and incorporating existent workflow (i.e., regular routine check-up) into the CVC program to get the work done at a decreased and efficient cost has been core to the process. These factors are all key considerations to ensure the successful implementation of digital technologies within the healthcare system (4, 25–27).

At the organizational and system levels, the IVR model was the outcome of a partnership with the industry in which the costs of the IVR and implementation were provided by an industry partner in exchange for the UOHI intellectual property for the algorithm design. Over 90% of the patients who have used the IVR application reported satisfaction with this type of monitoring. The positive evaluations of the e-health applications led to permanent MOHLTC funding. These strategies demonstrate how new innovations, through an evidence-informed approach, can evolve into institutionalized models that enable efficiency and improvement in patient care. Importantly managers must consider the potential for the sustainability of an innovation early on and ensure its alignment to the needs of all relevant stakeholders (12).

Table 1 summarizes the factors that were critical to the success of the CVC program, mapped against the factors reported in the literature. The organizational culture at the UOHI, availability of resources (i.e., financial, human, and leadership and management), technical features of the IT solutions, staff buy-in, perceived usability and usefulness of the technologies, staff training, value proposition, and perceived simplicity of the IT solutions all contributed to the successful spread and sustainability of the CVC program.

**Table 1:** Critical success factors in the CVC program innovation context.

Factors related to IT innovations failure in the literature	Experience of the CVC program innovation and critical success factors
Organizational culture (e.g., the environmental context of a healthcare organization, such as those that emphasize the need for continual improvement) (6, 8)	The UOHI aims to continually improve the care provided for cardiac patients. The organizational culture at the hospital creates an environment that emphasizes the importance of innovation and providing better care. Patient and family engagement is part of the engagement culture at the UOHI with patients or their caregiver serving on project teams and committees and contributing to the optimal decisions on innovations.
Structural factors (e.g., organizational characteristics, availability of resources, etc.) (6, 8)	Research and innovation funding was used to start the CVC program. Additional funding from the UOHI operating budget was also utilized to support the CVC program. Permanent funding through the MOHLTC was also secured for the program. In addition, given the large size of UOHI, the organization was better positioned to support the CVC program (e.g., more human resources, expertise available, etc.).
Technical challenges (e.g., lack of interoperability, limitations in the technical features of the IT solution, inability to adapt the systems to the needs of the user) (4, 9, 16)	The UOHI worked alongside a technology vendor (THM and IVR) to ensure that the features of the IT solution met the needs of the users (e.g., patients, nurses, etc.). Coordination and communication with the vendor was critical to ensure that technical challenges faced by staff members were addressed in a timely manner.
Capacity to support the IT solution (e.g., staffing) (4, 9)	Using the initial CVC program funding, and subsequent MOHLTC funding following the positive evaluation results, the UOHI was able to staff nurses to support the CVC program.
High levels of inertia (e.g., healthcare professional's resistance to change) (8)	The CVC program is “nurse-run” and involved the users from the initial project development. The users had ownership of the program, and were involved in various stages of the decision-making, including the selection of the initial technologies.
Limited Staff buy-in (e.g., due to changes in administrative and clinical workflows when implementing IT solutions) (7, 12, 41, 42)	While the IT solutions within the CVC program did require changes to staff members’ administrative and clinical workflow, there was high buy-in in the CVC program as staff perceived that the program, through its e-health applications, allowed them to remain connected with their patients and do their jobs more efficiently (e.g., seeing additional patients in a workday).

### 6.2 Research partnership and next steps

The continued evolution and adaptation of the CVC program to meet the needs of patients and demands on the hospital was supported by the collaborative work with research partners. These partnerships were instrumental in demonstrating the value of the various technological innovations, which led to established permanent funding, sustainability, and scalability of the innovation (Table 2).

**Table 2:** Findings from published studies related to the CVC program e-health applications at the UOHI.

Telemedicine
Study examining Healthcare and Education Access for Remote Residents by Telecommunications (HEARRT) (23) showed: • All first-time patients who were surveyed (*n* = 19) expressed satisfaction with their consultation, experienced no issues communicating with the consulting physician, and had confidence in the advice given during the consultation.
Telehome monitoring
Study on the impacts of THM in relation to accessibility, quality, and efficiency of healthcare (19) reported: • HF and angina patients (*n* = 105) with high risk of hospital readmission who were monitored with THM for three months experienced improved quality of life, found the equipment simple to use, and were highly satisfied with the care that they received **via** THM.
Study on the impacts after 3 months of THM (19) showed: • 51% decrease in admissions per patient for angina patients in THM group compared to usual care group at three months. • 61% reduction in number of days spent in hospital for angina patients in THM group compared to usual care group at three months. • 45% reduction in hospital admission rates for angina patients in THM group compared to usual care group at one year. • Angina patients in THM group made fewer emergency department visits compared to angina patients in usual care group at three months and at one year.
Study comparing THM for patients in rural vs. urban locations (35) showed: • Similar patterns of utilization of THM for rural and urban chronic heart failure patients in 2014. • The THM periods, the number of emergency visits, diuretic adjustments, and calls made by nurses did not vary based on geographic location (i.e., rural vs. urban).
Study on older adult's THM use (i.e., self-care practices, patient empowerment, and adoption factors) (28) reported: • Surveyed chronic HF patients (*n* = 23) perceived value in using telemonitoring, did not expect difficulty in using it, and did not experience barriers to adoption. • Observed improvement noted in patients’ confidence in their own ability to evaluate, rectify, and evaluate the effectiveness of the steps taken to rectify their HF symptoms. • Decreases were noticed over time in terms of self-care maintenance activities and capability to be involved in decision-making related to their chronic HF.
Interactive Voice Response
Study evaluating the use of IVR technology to increase medication compliance and reduce adverse health events (36) reported: • Increased compliance with medications for IVR group compared to usual care group. • Decreased emergency room visits and hospitalizations for IVR group compared to usual care group at six months.
Study on the use of an IVR system to improve survival of acute coronary syndrome (37) reported: • Increased compliance with medications by roughly 60% for IVR group compared to usual care group. • Decreased number of unplanned visits in IVR group compared to usual care group.
Study on the use of IVR by HF patients in relation to symptoms, compliance behaviour, lifestyle, and hospital readmission (38) reported: • Increase medication compliance in 12-week period. • Decreased symptom occurrence, weight gain, and hospital readmission rate in 12-week period.

THM, telehome monitoring; HF, heart failure; IVR, interactive voice response.

The UOHI continues to evolve and expand the CVC program. Starting as a technological innovation to enable access to care for diagnosis and post-discharge from the hospital, the CVC program is being leveraged to stabilize patients’ conditions before cardiac procedures for optimal care. One of the current on-going projects includes a scaleup of the THM for post operative surgery patients to monitor abnormal heart rhythms, incisions, and fluid balance. Funding was also secured to add pre-habilitation to the current post surgical IVR system, which allows for the virtual optimization of the condition of patients' pre-surgery, which can lead to faster recovery and better outcomes.

The IVR algorithms also continue to be developed to support the follow-up of arrhythmia patients waiting for their procedures, and open-heart surgery patients waiting for their operations. With the recent surge in artificial intelligence (AI) capabilities, remote monitoring too is expected to feature greater AI utilization. AI powered remote vital signs monitoring, physical activity monitoring, chronic disease monitoring, and emergency room monitoring have the potential to assist clinical decision making (30).

In summary, an early technological innovation (CVC program) was tested for feasibility through research using innovation funding. The early success demonstrated by research evidence enabled an integrated funded three-tiered comprehensive e-health care delivery model, which now provides services to patients with an array of complex cardiac conditions. Success, sustainability, and scale-up have been supported by adequate matching of patients with simple and intuitive technologies that cater to their needs, an agile approach enabling adaptation over time, and an opportunity for leveraging the infrastructure and institutional knowledge acquired through the three e-health applications to benefit a broader range of patients. As per Côté-Boileau and colleagues, innovation is “truly a journey” (8). The journey is not a straight line and can encounter unexpected events, which necessitate agility, continuous evaluation, and research partnerships that produce evidence to inform the adaptation and support the sustainability and scale-up of innovations.

## Data Availability

The original contributions presented in the study are included in the article/Supplementary Material, further inquiries can be directed to the corresponding author.
